# Pathological drivers of neurodegeneration in suspected non-Alzheimer’s disease pathophysiology

**DOI:** 10.1186/s13195-021-00835-2

**Published:** 2021-05-14

**Authors:** L. E. M. Wisse, R. de Flores, L. Xie, S. R. Das, C. T. McMillan, J. Q. Trojanowski, M. Grossman, E. B. Lee, D. Irwin, P. A. Yushkevich, D. A. Wolk, on behalf of the Alzheimer’s Disease NeuroImaging Initiative

**Affiliations:** 1 Department of Diagnostic Radiology, Lund University, Remissgatan 4, Room 14-520, 222 42 Lund, Sweden; 2 Penn Image Computing and Science Laboratory, Department of Radiology, University of Pennsylvania, Philadelphia, USA; 3 Penn Memory Center, Department of Neurology, University of Pennsylvania, Philadelphia, USA; 4 Université Normandie, Inserm, Université de Caen-Normandie, Inserm UMR-S U1237, GIP Cyceron, Caen, France; 5 Penn FTD Center, Department of Neurology, University of Pennsylvania, Philadelphia, PA USA; 6 Center for Neurodegenerative Disease Research, University of Pennsylvania, Philadelphia, PA USA

**Keywords:** Suspected non-Alzheimer’s pathophysiology, Neuropathologies diagnosis, Neurodegenerative pathologies, Hippocampus, Medial temporal lobe, Neurodegeneration, Primary age-related tauopathy, Limbic-predominant age-related TDP-43 encephalopathy

## Abstract

**Background:**

Little is known about the heterogeneous etiology of suspected non-Alzheimer’s pathophysiology (SNAP), a group of subjects with neurodegeneration in the absence of β-amyloid. Using antemortem MRI and pathological data, we investigated the etiology of SNAP and the association of neurodegenerative pathologies with structural medial temporal lobe (MTL) measures in β-amyloid-negative subjects.

**Methods:**

Subjects with antemortem MRI and autopsy data were selected from ADNI (*n*=63) and the University of Pennsylvania (*n*=156). Pathological diagnoses and semi-quantitative scores of MTL tau, neuritic plaques, α-synuclein, and TDP-43 pathology and MTL structural MRI measures from antemortem T1-weighted MRI scans were obtained. β-amyloid status (A+/A−) was determined by CERAD score and neurodegeneration status (N+/N−) by hippocampal volume.

**Results:**

SNAP reflects a heterogeneous group of pathological diagnoses. In ADNI, SNAP (A−N+) had significantly more neuropathological diagnoses than A+N+. In the A− group, tau pathology was associated with hippocampal, entorhinal cortex, and Brodmann area 35 volume/thickness and TDP-43 pathology with hippocampal volume.

**Conclusion:**

SNAP had a heterogeneous profile with more mixed pathologies than A+N+. Moreover, a role for TDP-43 and tau pathology in driving MTL neurodegeneration in the absence of β-amyloid was supported.

**Supplementary Information:**

The online version contains supplementary material available at 10.1186/s13195-021-00835-2.

## Background

In 2011, a framework for the preclinical stage of Alzheimer’s disease (AD) was proposed in which it was argued that β-amyloid deposition is followed by neurodegeneration and then subtle cognitive impairments [1]. In the context of this proposed sequence, individuals, including cognitively normal adults [2] and patients with mild cognitive impairment (MCI) [3], who display evidence of neurodegeneration but no β-amyloid deposition, fell into a category of suspected non-Alzheimer’s pathophysiology (SNAP). Similarly, in the new β-amyloid/tau/(neurodegeneration) (A/T/(N)) framework [4, 5], β-amyloid negative, tau negative, and neurodegeneration positive (A−T−N+) and A−T+N+ would be considered SNAP. The prevalence of SNAP is reported to be between 17 and 35%, both in cognitively impaired and unimpaired [6], with similar reported prevalences when applying A/T/(N) SNAP categories [7]. Adding to the clinical significance of this category, SNAP has been reported to show clinical and cognitive decline [8–10] and ongoing neurodegeneration [10, 11] compared to their biomarker-negative counterparts, though not in all studies [12–14]. Evidence for clinical progression has more frequently been reported in MCI-SNAP than cognitively normal SNAP individuals (CN-SNAP). However, the inconsistent findings on their clinical course may also be attributable to the heterogeneity in defining SNAP and the inherent heterogeneous nature of SNAP. In fact, the selection of the study population in extant studies likely affects the findings regarding the clinical course, demographics, and etiology of SNAP.

Regarding etiology, previous studies have revealed that SNAP individuals have increased cerebrovascular disease (CVD) [14, 15] and lower prevalence of APOE-ɛ4 carriers than their β-amyloid-positive counterparts [6], but the evidence regarding a role for subthreshold β-amyloid pathology has been inconsistent [14, 16]. While similarities between SNAP and primary age-related tauopathy (PART) have been pointed out [17] and ~23% of the A−N+ group was recently reported to be T+ [7], no evidence for elevated tau levels in SNAP was found in one recent relatively small study [18]. In conclusion, little is known about the heterogeneous etiology of SNAP, especially with regard to potential contributors that cannot be determined in vivo, such as TAR DNA-binding protein (TDP)-43, often associated with hippocampal sclerosis, α-synucleinopathy, and PART. Moreover, given the high prevalence of multiple pathologies in cognitively impaired, but also cognitively normal individuals [19, 20], it is likely that a considerable portion of SNAP individuals harbor more than one pathology.

We therefore aimed to investigate (1) the neuropathological diagnoses of SNAP compared to A+N+ and A−N− groups and (2) the association of neuropathology measures with structural measures of medial temporal lobe (MTL) subregions in β-amyloid-negative subjects. To encompass datasets with different proportions of non-AD clinical phenotypes, we examined data from both the ADNI cohort and the University of Pennsylvania Center for Neurodegenerative Disease Research (hereafter referred to as the UPenn dataset). Subjects in the ADNI cohort are relatively older and have a more amnesic phenotype, whereas subjects in the UPenn dataset are relatively younger and have a wider range of phenotypes.

## Methods

### Study population

*ADNI dataset*: Data used in the preparation of this article were obtained from the Alzheimer’s Disease Neuroimaging Initiative (ADNI) database (adni.loni.usc.edu). The ADNI was launched in 2003 as a public-private partnership, led by Principal Investigator Michael W. Weiner, MD. The primary goal of ADNI has been to test whether serial magnetic resonance imaging (MRI), positron emission tomography (PET), other biological markers, and clinical and neuropsychological assessment can be combined to measure the progression of mild cognitive impairment (MCI) and early Alzheimer’s disease (AD). For up-to-date information, see www.adni-info.org.

In ADNI, all subjects with available pathology data and a structural T1-weighted MRI at baseline were selected. See supplemental material for more information on ADNI. A total of 64 participants in ADNI had an available T1-weighted MRI at baseline and autopsy data available, of which 1 was excluded due to image quality, leaving 63 for the analyses.

*UPenn dataset*: Patient data were abstracted from the University of Pennsylvania Integrated Neurodegenerative Disease Database [21]. Patients selected were clinically evaluated and followed at the University of Pennsylvania’s Alzheimer’s Disease Core Center, Parkinson’s disease and Movement Disorder Clinic, Frontotemporal Degeneration Center, or the Michael J. Crescenz VA Medical Center’s Parkinson’s Disease Research, Education, and Clinical Center. For this study, subjects with neuromuscular disease (i.e., amyotrophic lateral sclerosis) or primarily a motor disorder (i.e., Parkinson’s disease) were excluded. We did not exclude patients with progressive supranuclear palsy (PSP) or corticobasal degeneration (CBD) as these diseases are also characterized by prominent cognitive symptoms. All patients with a research quality antemortem MRI were included in this study. A total of 207 participants had a research quality MRI and autopsy data available. Of these 207 participants, 45 had a neuromuscular disease or motor disorder (i.e., PD or ALS) and were excluded and 6 were excluded due to image or segmentation quality, leaving 156 participants for the analyses.

Both datasets are research cohorts and are recruiting participants from tertiary care centers. Where ADNI requires a more restricted phenotype of either cognitively normal older adults or patients with a clinical diagnosis of amnesic MCI or AD, the UPenn dataset includes clinically evaluated patients who agree to participate in research, but with a broader phenotype.

### Imaging protocol and image processing

*ADNI data*: The MRI scans were acquired from different scanners at multiple sites. Up-to-date information about MRI imaging protocols can be found at adni.loni.usc.edu/methods/mri-tool/mri-analysis. The resolution of the scans ranged from 0.94 × 0.94 × 1.2 to 1.25 × 1.25 × 1.2 mm^3^. The MRI at baseline was selected to capture SNAP at its earliest clinical phase to match prior work in ADNI.

*UPenn data*: In all patients, antemortem T1 structural MRI data was obtained, but a variety of protocols with resolutions ranging from 0.5 × 0. × 1 mm^3^ to 1.25 × 1.25 × 1.20 mm^3^ were performed. The MRI scan closest to the date of death was extracted.

### Automated segmentation of MTL subregions

**Fig. 1:**
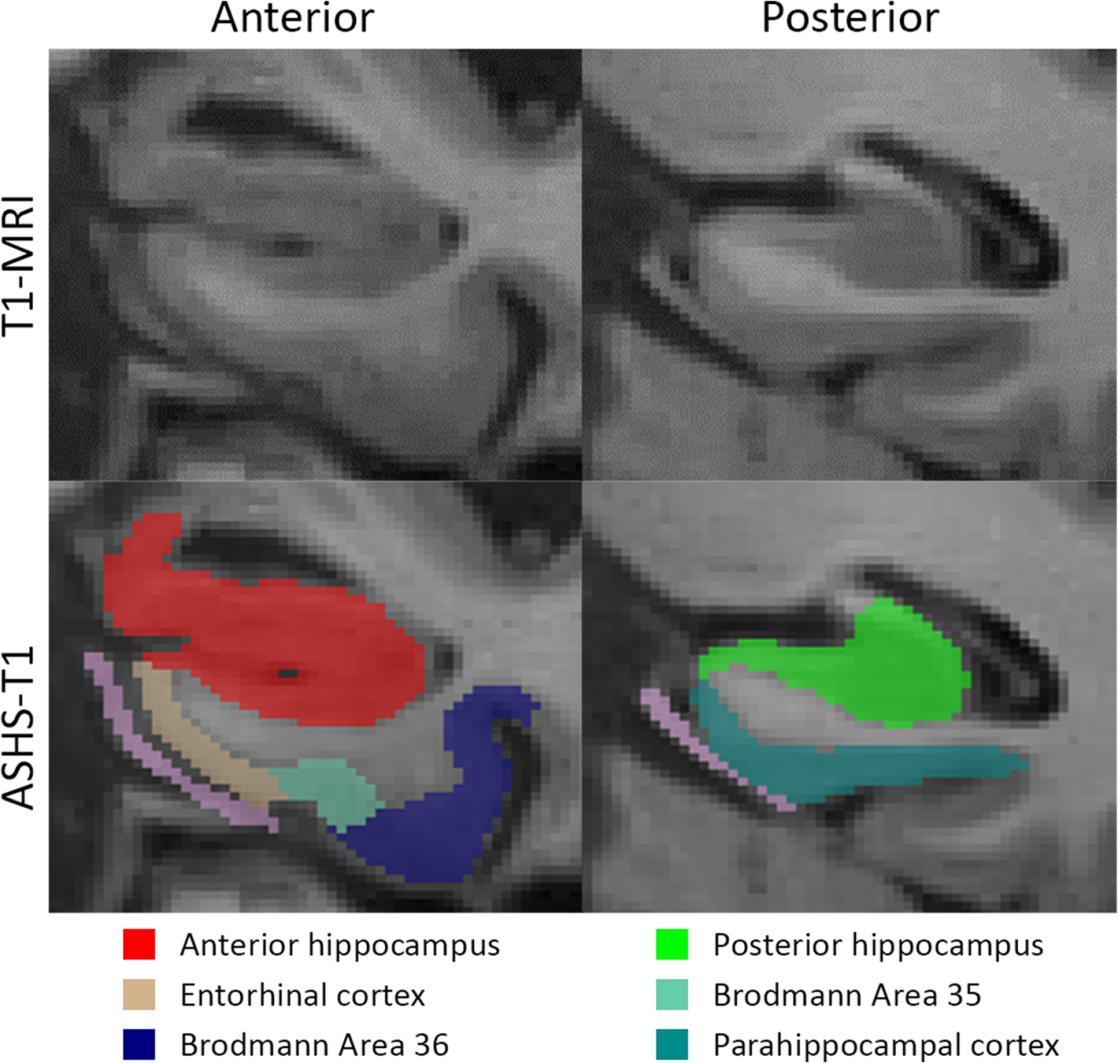
Example of an automated segmentation of medial temporal subregions using the automated segmentation of hippocampal subfields (ASHS)—T1 pipeline

MTL subregions were automatically segmented using the Automated Segmentation of Hippocampal Subfields (ASHS) package and a new T1-weighted segmentation pipeline (ASHS-T1) [22, 23]. Note that this new segmentation protocol offers the advantage of accounting for confounds of dural tissue and anatomic variation of the collateral sulcus. Six regions were segmented: the anterior and posterior hippocampus, the entorhinal cortex (ERC), Brodmann areas (BA) 35 and 36, and the parahippocampal cortex (PHC). An example of the ASHS-T1 segmentation is displayed in Fig. 1. Intracranial volume (ICV) was also measured using ASHS-T1 [23]. All segmentations were visually inspected. Failed segmentations were manually edited when feasible. Segmentations were excluded when the segmentation was clearly inaccurate and could not be edited because the borders could not be identified either because of poor image quality or too severe atrophy. In ADNI, hippocampal volumes for 1 subject were excluded. In the UPenn dataset, hippocampal volumes for 6 subjects were excluded. Volumes were analyzed for the hippocampal regions and thickness was obtained for the MTL cortical regions using a multi-template thickness analysis pipeline [23].

Cortical thickness measures for ERC, BA35, BA36, and PHC were used for the analyses investigating correlation with pathologies. For ADNI, the ERC, BA35, and BA36 were excluded for 4 subjects and PHC for 2 subjects based on image or segmentation quality in this region. For the UPenn dataset, the ERC was excluded for 21 subjects, BA35 for 16 subjects, BA36 for 17 subjects, and PHC for 9 subjects.

All MTL regions were averaged over the left and right hemispheres. Hippocampal volumes were corrected for ICV and age and thickness in MTL cortical regions for just age using the regression coefficients from a separate group of 184 β-amyloid-negative cognitively normal older adults from ADNI-GO/2 (mean age 71.7±6.1 years). β-amyloid status was determined based on the standard cutoff of SUVR ≥1.11 using florbetapir PET scans [24].

### β-amyloid and neurodegeneration status

The neurodegeneration cutoff was obtained by taking the 90th percentile of hippocampal volumes of β-amyloid-positive AD patients at baseline from ADNI-GO/2. β-amyloid status was determined based on the standard cutoff of SUVR ≥1.11 using florbetapir PET scans [24]. Taking the 90th percentile of hippocampal volumes, or other neurodegeneration measures of β-amyloid-positive AD patients is a common approach to emphasize sensitivity, but potentially at the cost of specificity [2, 3, 25]. Note that β-amyloid status was only determined by PET to correct MTL structural measures for ICV and age (previous section) and to determine neurodegeneration cutoff.

To make our study comparable to previous literature, we initially aimed to determine β-amyloid status using in vivo measures. However, in vivo biomarkers of β-amyloid were not available in all participants close to the time of the MRI scan. We therefore chose a pathology cutoff based on the detection level of PET [26] and based β-amyloid status on a CERAD score ≥B for β-amyloid positivity (A+; A+/− only refers to β-amyloid status determined by the CERAD score). This is a conservative cutoff for A− cases, as, if anything, amyloid levels would be expected to be lower at the time of the in vivo MRI scan. All analyses presented in the “Results” section utilize the β-amyloid status based on the CERAD score.

To further confirm how well β-amyloid status based on the CERAD score matches that of β-amyloid PET, which is often used to determine SNAP, we compared the cutoff of the CERAD score to the standard cutoff for the florbetapir scan for 30 subjects in the ADNI dataset for whom a florbetapir scan was available. The scan at the latest available timepoint was selected. The two cutoffs showed an agreement of 87.6% (3 subjects with a positive β-amyloid PET scan were β-amyloid negative based on the CERAD cutoff, and 1 subject with a negative β-amyloid PET scans was β-amyloid positive based on the CERAD cutoff), see Supplementary Table 1.

### Neuropathological diagnoses and neuropathology measures

For both datasets, the number of neuropathological diagnoses for each individual was counted. Note that for ADNI up to five different neuropathological diagnoses were provided based on convention, while there were up to three for the UPenn dataset. Additionally, for each of the studies, AD neuropathologic change (ADNC) was established according to the criteria of Montine et al. [27]. Intermediate to high ADNC was taken as the presence of AD. Primary age-related tauopathy (PART) was established based on a CERAD score of 1 or lower (“possible” and “definite” PART, respectively) and a Braak score of 4 or lower [28].

*ADNI data*: All autopsies were performed at the respective site. Most brains were fixed with formalin, except for two which were fixed with paraformaldehyde. The tau-antibody used was PHF-1 in 61 cases and a non-phospho-specific tau stain in 1 case. The β-amyloid antibody was 10DS for all cases and the α-synuclein antibody was a phospho-specific (e.g., pSYN#64) one for all cases. The TDP-43 antibody was a phospho-specific one except for one case where it was a non-phospho-specific one. ABC scores were established using the NIA-AA protocol [27]. The scoring system for β-amyloid neuritic plaques, tau, α-synuclein Lewy bodies (LB), and TDP-43 neurocytoplasmatic inclusion score is shown in Supplementary Table 2. As the present study specifically focused on the MTL, a composite score was calculated by averaging the scores of CA1, ERC, and amygdala for each lesion of interest (tau, β-amyloid, TDP-43, and α-synuclein). MTL data for β-amyloid, tau, and α-synuclein was missing in one case and for TDP-43 in two cases.

*UPenn data*: All autopsies were performed at the Penn Center for Neurodegenerative Disease Research (CNDR). Thirteen regions are routinely examined in the CNDR neuropathology evaluations as described in previous publications [21]. More precisely, tissue was embedded in paraffin blocks and cut into 6-μm sections for immunohistochemistry using the following primary antibodies: NAB228 (monoclonal antibody [mAb], 1:8000, generated in the CNDR) to detect β-amyloid deposits, phosphorylated tau PHF-1 (mAb, 1:1000, a gift from Dr. Peter Davies) to detect phosphorylated tau deposits, TAR5P-1D3 (mAb, 1:500, a gift from Dr. Manuela Neumann and Dr. E. Kremmer) to detect phosphorylated TDP-43 deposits, and Syn303 (mAb, 1:16,000, generated in the CNDR) to detect the presence of pathological conformation of α-synuclein. Each region was assigned a semi-quantitative score, i.e., none (0), rare (0.5), mild (1), moderate (2), or severe (3) for individual lesions (tau, neuritic plaques, TDP-43, and α-synuclein pathologies). Similar as for the ADNI data, a composite score for the MTL was calculated by averaging the scores of CA1/subiculum, ERC, and amygdala for each lesion of interest (tau, β-amyloid, TDP-43, and α-synuclein). MTL data for β-amyloid and tau was missing in three cases, for α-synuclein in two cases, and for TDP-43 in six cases.

Note that in both datasets the tau pathology score does not only reflect neurofibrillary tangles (NFT) but represents multiple conformations of tau and is therefore referred to as tau and not NFT.

### Statistical analyses

We compared SNAP with the A−N− and the A+N+ groups (to minimize the number of comparisons, we did not analyze the A+N− group in this study). Demographics, number, and presence of neuropathological diagnoses were compared with a Mann-Whitney *U* test for continuous and Fisher’s exact test for categorical variables. Only the neuropathological diagnoses with the highest prevalence were analyzed in comparisons of diagnostic categories to limit the number of analyses.

The association of MTL MRI measures with MTL pathology measures in the A− group was assessed using a Spearman correlation, corrected for time between MRI and death and sex using the R package “ppcor.” All tests were two-tailed. Because autopsy data is so rare, we used a liberal detection threshold of *p*=0.05 two-tailed for all tests.

## Results

### Demographics

*ADNI dataset:* 22.2% of the participants met the criteria for SNAP (Table 1). The groups were not different in terms of age, but the SNAP group had a longer time interval between MRI and date of death than A+N+ at a trend level and a larger proportion of males than A+N+ and A−N− at a trend level.

**Table 1:** Demographics of the ADNI and UPenn datasets

	ADNI			UPenn		
	A−N−	SNAP	A+N+	A−N−	SNAP	A+N+
Number (%)	6 (9.5)	14 (22.2)	35 (55.6)	11 (7.1)	47 (30.1)	76 (48.7)
Age at death (years)	84.5±3.8	83.6±8.4	82.0±6.9	68.6±5.9	68.9±9.8	74.2±11.8*
Gender (% male)	4 (66.7)^◊^	14 (100)	27 (77.1)^◊^	8 (72.7)	47 (61.8)	26 (55.3)
Time difference between MRI and date of death (years)	4.5±2.1	5.9±2.8	4.3±2.5^◊^	2.1±2.5	2.1±2.0	3.2±2.5*
Clinical diagnosis at MRI						
Control (%)	4 (66.7)	1 (7.1)	2 (5.7)	0 (0)	0 (0)	0 (0)
MCI (%)	2 (33.3)	11 (78.6)	16 (45.7)	0 (0)	1 (2.1)	6 (7.9)
Dementia (%)	0 (0)	2 (14.3)	17 (48.6)	11 (100)	46 (97.9)	69 (90.8)
Others (%)	0 (0)	0 (0)	0 (0)	0 (0)	0 (0)	1 (1.3)^a^
Latest clinical diagnosis						
Control (%)	3 (50.0)	1 (7.1)	0 (0)	0 (0)	0 (0)	0 (0)
MCI (%)	2 (33.3)	2 (14.3)	4 (11.4)	0 (0)	1 (2.1)	4 (5.3)
Dementia (%)	1 (16.7)	3 (78.6)	31 (88.6)	11 (100)	46 (97.9)	72 (94.7)

^◊^<0.10; *p<0.05 for comparison with SNAP. ^a^This case had a clinical diagnosis of Parkinson’s disease but a neuropathological diagnosis of progressive supranuclear palsy. *SNAP* suspected non-Alzheimer’s pathophysiology, *A* β-amyloid, *N* neurodegeneration, *MCI* mild cognitive impairment

*UPenn dataset:* 30.1% of the participants met the criteria for SNAP (Table 1). The groups were not different in terms of gender, but the A+N+ group was significantly older than SNAP and had a significantly longer time interval between MRI and date of death.

Clinical diagnosis at MRI scan and latest clinical diagnosis can also be observed in Table 1. Qualitatively, it can be observed that the groups in the UPenn dataset are more severely impaired with a larger percentage of dementia and no cognitively normal individuals, as compared to the ADNI dataset.

### Neuropathological diagnoses

**Fig. 2:**
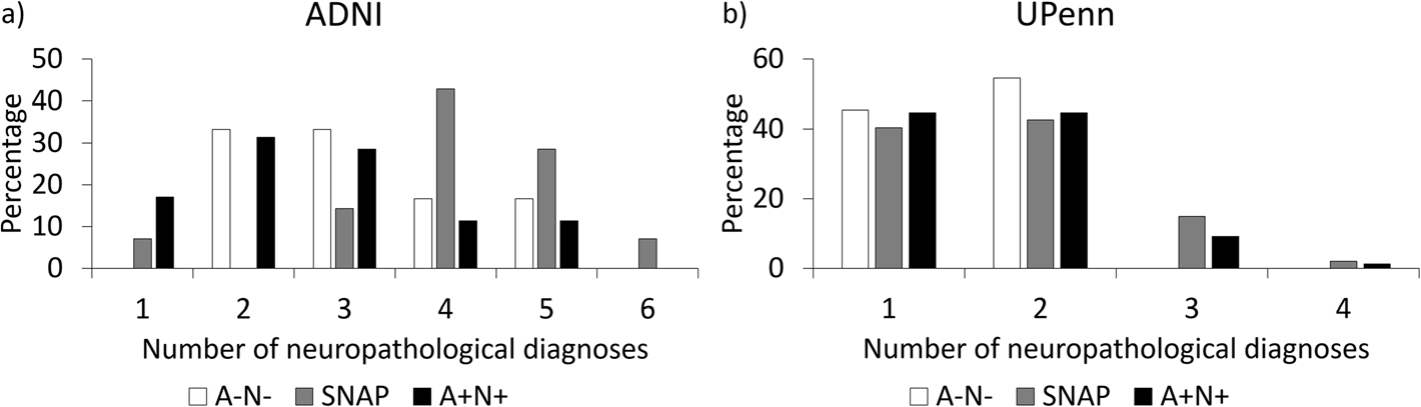
Prevalence of the number of neuropathological diagnoses in SNAP, A−N−, and A+N+ in the ADNI (**a**) and UPenn (**b**) datasets. SNAP, suspected non-Alzheimer’s pathophysiology; A, β-amyloid; N, neurodegeneration

**Fig. 3:**
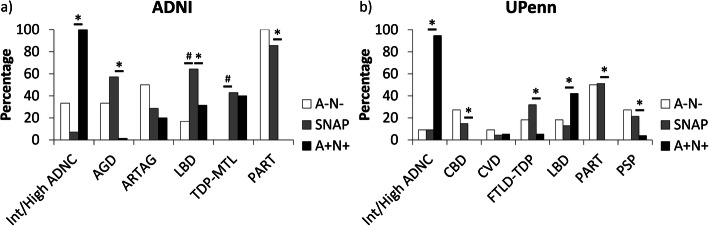
Prevalence on neuropathological diagnoses in SNAP, A−N−, and A+N+ in the ADNI (**a**) and UPenn (**b**) datasets. Note that the raw values are displayed in Supplementary Table 3. ^#^<0.10; *p<0.05. SNAP, suspected non-Alzheimer’s pathophysiology; A, β-amyloid; N, neurodegeneration; ADNC, AD neuropathological change; AGD, argyrophilic grain disease; ARTAG, aging-related tau astrogliopathy; LBD, Lewy body disease; TDP-MTL, TAR DNA-binding protein in the Medial Temporal Lobe; PART, primary age-related tauopathy; CBD, corticobasal degeneration; CVD, cerebrovascular disease; FTLD-TDP, frontotemporal lobar degeneration with TDP-43 inclusions; PSP, progressive supranuclear palsy

*ADNI dataset:* Despite similar age, the SNAP group had a higher number of different neuropathological diagnoses per individual than A+N+ (*p*<0.001) but not A−N− (*p*=0.13; Fig. 2a). When analyzing the specific neuropathological diagnoses, it can be observed that, qualitatively, SNAP had a high prevalence of primary age-related tauopathy (PART), TDP-MTL (which would likely be classified as limbic-predominant age-related TDP-43 encephalopathy, or LATE, with new criteria [29]), argyrophilic grain disease (AGD), and Lewy body disease (LBD) (Fig. 3a). Compared to A+N+, SNAP had a significantly higher prevalence of AGD, LBD, and PART, as well as a significantly lower prevalence of AD.

*UPenn dataset:* There was no significant difference between the number of pathological diagnoses in each of the groups (Fig. 2b). Qualitatively, SNAP had a relatively high prevalence of frontotemporal lobar degeneration with TDP-43 inclusions (FTLD-TDP), PART, and progressive supranuclear palsy (PSP; Fig. 3b). There were no significant differences between SNAP and A−N− in the prevalence of any of the diagnostic categories. The A+N+ group had a significantly higher prevalence of AD, by definition, and LBD. SNAP had a significantly higher prevalence of corticobasal degeneration (CBD), FTLD-TDP, PART, and PSP than A+N+.

### The association of neurodegenerative pathologies with MTL structural measures

**Fig. 4:**
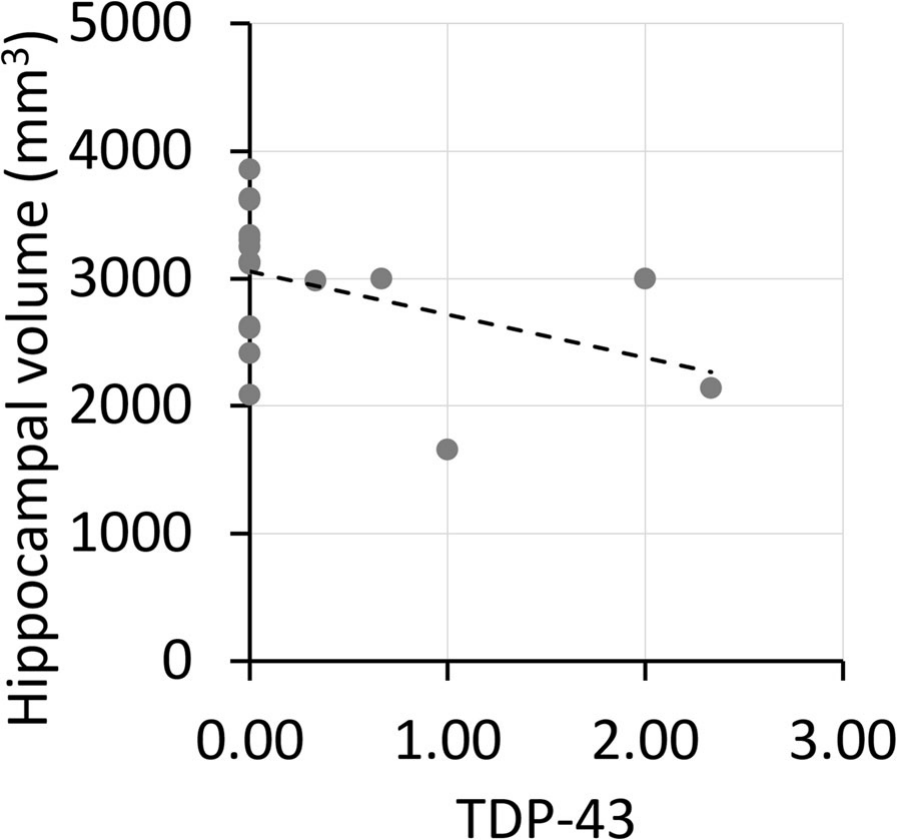
Scatterplot of the association of TDP-43 with hippocampal volume in β-amyloid-negative individuals in ADNI. TDP, TAR DNA-binding protein

*ADNI dataset:* No significant associations were found between the neurodegenerative pathologies and MTL structural measures in the β-amyloid-negative individuals (Table 2). However, at a trend level, a higher TDP-43 score was associated with smaller hippocampal volumes (Fig. 4). It should be noted that only five participants had a TDP-43 score higher than 0, warranting caution in interpreting these results.

**Table 2:** The association of neurodegenerative pathologies in the MTL with MTL volume/thickness measures in β-amyloid negative individuals. All pathologies are entered in one model, with time between scan and death and gender as covariates. Spearman rank values are reported in the table

	Whole Hippo	Ant Hippo	Post Hippo	ERC	BA35	BA36	PHC
**ADNI**							
NP	−0.11	−0.12	0.08	0.14	−0.13	−0.14	−0.31
Tau	0.02	0.09	−0.17	−0.31	−0.11	0.08	0.05
α-synuclein	0.00	−0.08	0.35	0.23	0.22	−0.08	0.26
TDP-43	−0.48^◊^	−0.40	−0.41	0.00	0.14	0.57^◊^	0.49
**UPenn**							
NP	0.16	0.14	0.13	0.20	0.17	0.09	0.32*
Tau	−0.40**	−0.34*	−0.46***	−0.34*	−0.31*	−0.29^◊^	−0.08
α-synuclein	−0.10	−0.03	−0.13	−0.11	−0.14	−0.16	−0.15
TDP-43	−0.30*	−0.33*	−0.26^◊^	−0.13	−0.25^◊^	−0.22	−0.11

^◊^*p*<0.10, *p<0.05, **p<0.01, ***p<0.001. *NP* neuritic plaques, *TDP* TAR DNA-binding protein, *Ant* anterior, *Post* posterior, *ERC* entorhinal cortex, *BA* Brodmann area, *PHC* parahippocampal cortex

**Fig. 5:**
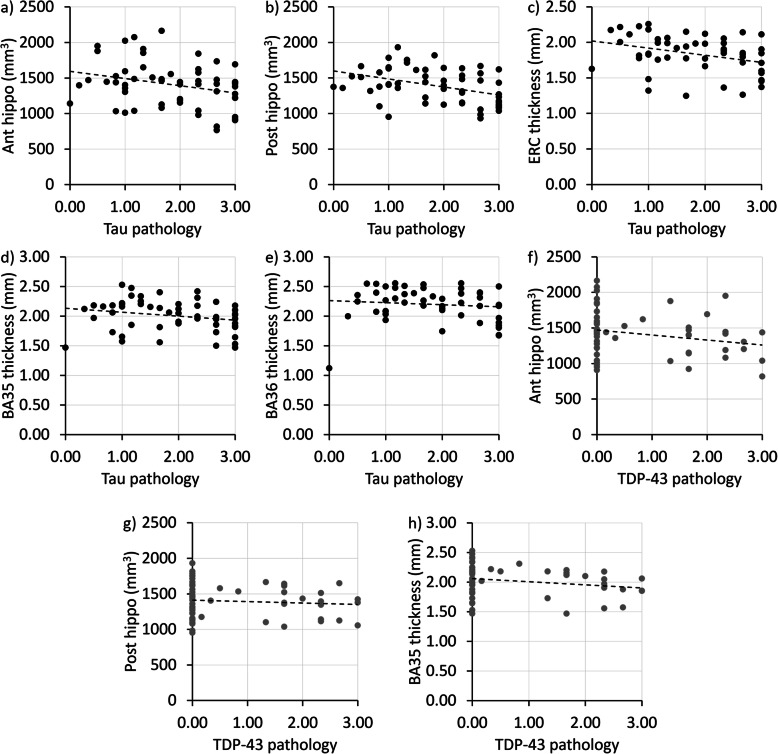
Scatterplot of the association of Tau (**a**–**e**, black dots) and TDP-43 pathology (**f**–**h**, grey dots) with medial temporal lobe structural measures in β-amyloid negative individuals in the UPenn dataset. TDP, TAR DNA-binding protein; ERC, entorhinal cortex; BA, Brodmann area

*UPenn dataset:* In the β-amyloid-negative individuals, a higher TDP-43 pathology score was significantly associated with smaller anterior hippocampal volumes and total hippocampal volumes (Table 2). Moreover, a higher tau pathology score was significantly associated with smaller anterior and posterior hippocampal volumes and ERC and BA35 thickness (Fig. 5). A higher neuritic plaque score was significantly associated with larger PHC thickness. This is likely a spurious finding given that this is in the opposite direction from what is expected and because a (nonsignificant) negative correlation of the same magnitude is observed in the ADNI dataset.

## Discussion

In this study, we found support that SNAP reflects a heterogeneous group of pathological diagnoses, including FTLD-TDP, PSP, LBD, PART, TDP-MTL, and AGD. Moreover, in the ADNI dataset, SNAP individuals had a significantly greater number of different neuropathological diagnoses per individual than A+N+. In the second part of this study, we investigated what pathologies drive neurodegeneration in the MTL in the absence of β-amyloid. We found associations of tau pathology with hippocampal regions, ERC, and BA35 and of TDP-43 pathology with hippocampal volume in the Penn dataset and for TDP-43 at a trend level in the ADNI dataset.

### Neuropathological diagnoses

One of the major findings of this study is that individuals with SNAP had a greater number of co-existing neuropathological diagnoses than A+N+ in ADNI, suggesting a more mixed profile in SNAP. Potentially, individuals with SNAP have generally a more indolent course and reflect the accumulation of multiple pathologies associated with aging as the driver of their neurodegeneration. Indeed, SNAP was slightly older than A+N+ in the ADNI dataset, but this difference did not reach statistical significance. It should be noted that we did not observe a higher number of diagnoses in SNAP in the UPenn dataset. We hypothesize that this difference stems from the fact that the UPenn dataset is a more diverse cohort with regard to inclusion of individuals with non-AD phenotypes and, thus, SNAP may reflect more aggressive non-AD proteinopathies (e.g., FTLD spectrum disorders) than ADNI. The age difference may also play a role, as individuals who have neurodegeneration at a younger age, and, thus, are younger at death, would be less likely to accrue other age-related pathologies [30]. Age might therefore also be an important factor when considering the etiology of SNAP. No significant difference in the number of diagnoses was found between SNAP and A−N− in either of the datasets. This may partly be due to a lack of power due to the small sample size of the A−N− groups, especially in ADNI where in absolute terms the number of diagnoses in A−N− was lower than SNAP. In the UPenn dataset, another explanation may also be that we used hippocampal volume as a neurodegeneration marker, where in this dataset with a broad range of phenotypes neurodegeneration may actually be more prominent in other brain regions. It is therefore possible that a number of A−N− were “misclassified” as N−.

Additionally, we found support that AD is not the cause of SNAP in the majority of the cases, a hypothesis that was included in the naming of this group. Specifically, we found support for a higher prevalence of AGD, LBD, PART, and TDP-MTL, likely LATE, in the ADNI dataset and CBD, FTLD-TDP, PSP, and PART in the UPenn dataset. It should be noted that the results regarding the lower prevalence of AD (and also a higher prevalence of PART) in SNAP are somewhat circular, as SNAP was defined by low CERAD score, and, thus, was less likely to be considered intermediate or high probability of ADNC. That said, the CERAD score cutoff at autopsy was largely consistent with in vivo measurement of β-amyloid status with PET so the majority of SNAP cases would likely carry that designation if based on in vivo biomarkers rather than neuritic plaque burden at autopsy. Thus, this does not support the notion that SNAP just reflects individuals on the AD continuum with subthreshold amyloid. These findings line up with previous studies of neuropathological diagnoses in SNAP reporting a low prevalence of AD [31, 32] but supporting the presence of PART, AGD, and LBD [32, 33]. Importantly, there is a clear difference in the neuropathological diagnoses in the ADNI and UPenn datasets indicating that the selection of the dataset contributes much to underlying etiology and potentially the heterogeneous results observed in previous studies with regard to longitudinal neurodegeneration and cognitive decline [8, 10–14].

In the context of the AT(N) framework [4, 5], this means that some SNAP individuals can indeed be categorized as A−T+(N+). However, it is likely that comorbid pathologies, besides tau pathology, may also contribute to the neurodegeneration observed in this group. The A−T−(N+), as expected, is very heterogeneous.

### The association of neurodegenerative pathologies with MTL structural measures

In the UPenn dataset, we found an association of tau pathology with hippocampal volumes, entorhinal, and BA35 thickness. While the tau pathology score in this dataset represents multiple confirmations of tau, the specific association with MTL regions involved in early Braak stages and the high prevalence of PART in the β-amyloid-negative group suggests that NFT pathology is partly driving this association. Indeed, previous papers have also found support for an association of Braak stages of NFT pathology and MTL atrophy in PART [34, 35]. The lack of an association of NFT pathology with MTL measures in the absence of β-amyloid in the ADNI dataset is puzzling. Potentially, this is due to a lack of power as the correlation for at least one region, ERC, was of a magnitude within the range of the Penn cohort (*r*=−0.31). On the other hand, it should be noted that the MTL NFT scores were virtually the same in A−N− and SNAP individuals (data not shown). Perhaps the range of the NFT score was too limited to detect an association. It is surprising though that the A−N− group had such high levels of NFT pathology without significant neurodegeneration in the hippocampus. A possible explanation is that there is a lag between the accumulation of NFT pathology and neurodegeneration and the A−N− group may not have had a significant load of NFT pathology for a long enough time to develop neurodegeneration. Another possible explanation is that these individuals are somehow resistant to the effects of NFT pathology, as a recent study indicated reporting a number of cases with Braak II-III NFT pathology but still healthy appearing neurons in the MTL and also limited neurodegeneration in the neocortex [36].

Both studies on the other hand showed an association of TDP-43 pathology with hippocampal volumes (although only at a trend level in ADNI), whereas in the UPenn dataset the association with anterior hippocampal volume also reached significance (however, the difference in correlation with the posterior hippocampus was only marginal). The degree to which TDP-43 can be divided into cases of FTLD-TDP versus LATE remains controversial [37, 38]. Notwithstanding this issue, previous literature indicates that TDP-43 has been found to be associated with volume loss in the MTL [39–41], both in datasets including and excluding cases which would meet common pathological definitions of FTLD. However, two recent papers found no support for an association of TDP-43 pathology with hippocampal volumes in the absence or low likelihood of Alzheimer’s dementia [42, 43], again both in datasets including and excluding FTLD brains. This seems in contrast with our findings; however, in the UPenn dataset, a larger portion of the cases of TDP-43 pathology may be FTLD-TDP-related, or at least these cases had more typical phenotypic features of FTD, which may be driving our findings. Indeed, when the FTLD-TDP cases are removed from the analyses, there is no longer an association between TDP-43 pathology and hippocampal volumes (data not shown). On the other hand, the trend-level association of TDP-43 with hippocampal volumes in the ADNI dataset, which some would consider as LATE (only one case had FTLD-TDP) combined with the high prevalence of the neuropathological diagnosis of TDP-MTL, provides some evidence for a role for LATE as a driver of neurodegeneration in the absence of β-amyloid pathology and as one of the causes of SNAP.

### Limitations and strengths

A limitation of the current study is the small sample size, especially for the A−N− groups, which likely limited the power to detect differences with the SNAP group. Relatedly, especially in the UPenn dataset, a significant number of the segmentations were excluded (between 5.8 and 13.4%) for the extrahippocampal regions due to image quality or severe atrophy further limiting the sample size for certain analyses. Additionally, as for all studies associating antemortem MRI with autopsy information, our findings may have been diluted by the time interval between the MRI scan and the time of autopsy. Finally, we used semi-quantitative scores rather than quantitative measures of pathology burden which were obtained from only one hemisphere. This may have further limited our ability to detect associations between neurodegenerative pathologies and structural MRI measures.

A strength of this paper is the linkage of neurodegenerative pathologies to antemortem MRI, and especially neurodegenerative pathologies for which currently no in vivo biomarkers are available. Another strength is the use of a newly developed and robust method for measuring granular MTL subregional measures. Finally, an important strength of the current study is the inclusion of two datasets, including patients with different phenotypes and of different ages. The discrepant findings in two different datasets allowed us to highlight that the selection of dataset is an important factor in driving the study findings, where both age and phenotype may influence what factors may drive neurodegeneration and what neuropathological diagnoses may underly SNAP.

## Conclusion

In this unique study leveraging a robust T1 pipeline for MTL segmentation and two different antemortem MRI/postmortem pathology datasets, we found further support that SNAP has a heterogeneous, mixed profile with neuropathological diagnoses such as LBD, AGD, TDP-MTL, PART, FTLD-TDP, and PSP, which may be dependent on the selection of the study population. Finally, we found initial evidence for a role of TDP-43 and tau pathology as drivers of neurodegeneration in the absence of β-amyloid.

## Supplementary Information

**Additional file 1:** Supplementary methods for ADNI. **Supplementary Table 1.** Comparison of the CERAD cut-off and florbetapir cut-off to determine β-amyloid status. β-amyloid negativity for CERAD score was determined by a score of 0 or A, and positivity by a score of B or C. **Supplementary Table 2.** Scoring system of pathologies in the different regions in ADNI. **Supplementary Table 3.** Prevalence of neuropathological diagnoses in SNAP, A-N- and A+N+ in the ADNI (a) and UPenn (b) dataset.

## Data Availability

ADNI data is publicly available. Moreover, tissue from the University of Pennsylvania Integrated Neurodegenerative Disease Database is available upon request.
