# Supplementary material for: Pathological drivers of neurodegeneration in suspected non-Alzheimer’s disease pathophysiology
Source: Alzheimers Res Ther. 2021 May 14;13:100. doi: 10.1186/s13195-021-00835-2 (PMC8122549; doi:10.1186/s13195-021-00835-2)
Supplement: Supplementary file 1 — Additional file 1: Supplementary methods for ADNI. Supplementary Table 1. Comparison of the CERAD cut-off and florbetapir cut-off to determine β-amyloid status. β-amyloid negativity for CERAD score was determined by a score of 0 or A, and positivity by a score of B or C. Supplementary Table 2. Scoring system of pathologies in the different regions in ADNI. Supplementary Table 3. Prevalence of neuropathological diagnoses in SNAP, A-N- and A+N+ in the ADNI (a) and UPenn (b) dataset. [file 13195_2021_835_MOESM1_ESM.docx]

**Supplementary Material**

*ADNI study*

Data used in the preparation of this article were obtained from the Alzheimer's Disease Neuroimaging Initiative (ADNI) database ([adni.loni.usc.edu](https://ida.loni.usc.edu/collaboration/access/adni.loni.usc.edu)). The ADNI was launched in 2003 as a public-private partnership, led by Principal Investigator Michael W. Weiner,MD. The primary goal of ADNI has been to test whether serial magnetic resonance imaging (MRI), positron emission tomography (PET), other biological markers, and clinical and neuropsychological assessment can be combined to measure the progression of mild cognitive impairment (MCI) and early Alzheimer's disease (AD). For up-to-date information, see [www.adni-info.org](https://ida.loni.usc.edu/collaboration/access/www.adni-info.org).

**Supplementary Table 1.** Comparison of the CERAD cut-off and florbetapir cut-off to determine β-amyloid status. β-amyloid negativity for CERAD score was determined by a score of 0 or A, and positivity by a score of B or C.

The case who is A+ according to the CERAD but A- according to the Florbetapir scan, has a CERAD score of C, Thal phase of 5 and Braak stage of V and approximately one year between the Florbetapir scan and death.

The three cases who are A- according to the CERAD but A+ according to the Florbetapir scan all had a Thal score of 4 or higher, but a CERAD score of 0 (n=1) or 1 (n=2).

|  |  | CERAD | |
| --- | --- | --- | --- |
|  |  | A- | A+ |
| Florbetapir | A- | 9 | 1 |
|  | A+ | 3 | 17 |

CERAD=Consortium to Establish a Registry for Alzheimer's Disease; A=β-amyloid; N=neurodegeneration

**Supplementary Table 2.** Scoring system of pathologies in the different regions in ADNI

| **Score** | **Neuritic plaque** | **Neurofibrillary tangles** | **α-synuclein** | **TDP-43** |
| --- | --- | --- | --- | --- |
| 0 | None | None | None | None |
| 1 | 1-5 NP/Tau/Abeta plaques/1mm2 | 1-5 NP/Tau/Abeta plaques/1mm2 | < 1 LB/TDP-43 inclusion per x10 field | < 1 LB/TDP-43 inclusion per x10 field |
| 2 | > 6 < 20 | > 6 < 20 | 1-3 LB/TDP-43 | 1-3 LB/TDP-43 |
| 3 | > 20 | > 20 | 4-10 LB/TDP-43 | 4-10 LB/TDP-43 |
| 4 | - | - | =>10 or numerous LB/TDP-43 inclusions | =>10 or numerous LB/TDP-43 inclusions |

TDP= TAR DNA-binding protein (TDP); NP=Neuritic plaques; LB=Lewy Bodies

**Supplementary Table 3.** Prevalence on neuropathological diagnoses in SNAP, A-N- and A+N+ in the ADNI (a) and UPenn (b) dataset. This table displays the raw values depicted in Figure 3 in the main manuscript.

|  | **ADNI** | | |
| --- | --- | --- | --- |
|  | **A-N-** | **SNAP** | **A+N+** |
| Int/High ADNC (%) | 33.3 | 7.1 | 100 |
| AGD (%) | 33.3 | 57.1 | 1.4 |
| ARTAG (%) | 50 | 28.6 | 20.0 |
| TDP-MTL (%) | 0 | 42.9 | 40.0 |
| PART (%) | 100 | 85.7 | 0 |
|  | **UPenn** | | |
|  | **A-N-** | **SNAP** | **A+N+** |
| Int/High ADNC (%) | 9.1 | 9.1 | 94.7 |
| CBD (%) | 27.3 | 14.9 | 0 |
| CVD (%) | 9.1 | 4.3 | 5.3 |
| FTLD-TDP (%) | 18.2 | 31.9 | 5.3 |
| LBD (%) | 18.2 | 12.8 | 42.1 |
| PART (%) | 50.0 | 51.2 | 0 |
| PSP (%) | 27.3 | 21.3 | 3.9 |

SNAP=Suspected non-Alzheimer’s pathophysiology: A=β-amyloid; N=neurodegeneration; ADNC=AD neuropathological change; AGD=Argyrophylic grain disease; ARTAG=Aging-Related Tau Astrogliopathy; LBD=lewy body disease; TDP-MTL=; PART=Primary Age-Related Tauopathy; CBD=Corticobasal degeneration; CVD=Cerebrovascular disease; FTLD-TDP=Frontotemporal Lobar Degeneration with TDP-43 inclusions; PSP=Progressive Supranuclear palsy
